# Mycelium-Based Composites for Interior Architecture: Digital Fabrication of Acoustic Ceiling Components

**DOI:** 10.3390/biomimetics10110729

**Published:** 2025-11-01

**Authors:** Müge Özkan, Orkan Zeynel Güzelci

**Affiliations:** 1 Graduate School, Istanbul Technical University, Istanbul 34496, Türkiye; 2 Faculty of Architecture, Istanbul Technical University, Istanbul 34367, Türkiye

**Keywords:** interior architecture, biodesign, biomaterials, digital fabrication, sustainable design, mycelium-based composites, acoustic materials

## Abstract

This study examines the integration of digital fabrication technologies into the design and production of mycelium-based components, addressing the growing demand for sustainable and innovative interior design solutions. Using a parametric design approach, modular and customized suspended ceiling elements were developed for a specific interior setting to explore a material-specific design approach for mycelium-based components. Three-dimensional printing was employed to produce molds, which were subsequently tested with plaster, silicone, and mycelium across three different scales. Experimental observations focused on the overall form, surface details, growth behavior and dimensional accuracy, systematically capturing volumetric deviations arising from the living nature of the material. In parallel, acoustic performance was evaluated through simulations using the Sabine method. The untreated condition demonstrated the longest reverberation times, whereas conventional panels achieved reductions consistent with typical comfort standards. Prototypes produced with mycelium yielded measurable decreases in reverberation time compared to the untreated condition, particularly within the speech frequency range, and approached the performance of standard acoustic panels. These findings suggest that mycelium-based components, when further optimized in terms of density and geometry, hold the potential to contribute both aesthetic and acoustic value within sustainable interior environments.

## 1. Introduction

The construction sector causes serious and often irreversible damage to ecosystems. With the rapid advancement of production technologies, this sector has become one of the highest consumers of both renewable and non-renewable natural resources. This has led to significant environmental problems, including uncontrolled waste generation, high carbon emissions, and resource depletion [1]. According to World Bank data, the global population has increased sevenfold over the past two centuries [2]. This demographic growth has accelerated construction activities to meet housing demands, directly positioning the construction sector as one of the primary contributors to global carbon emissions. Conventional building materials, such as concrete, steel, and fired clay bricks, which have energy-intensive production processes and limited recyclability, significantly contribute to these impacts through their production, transportation, and disposal processes.

In recent years, sustainable design approaches have created the need to reassess the interaction of traditional building systems with the environment. In this context, bio-based composite materials have been explored as sustainable alternatives to reduce the environmental footprint of the construction sector. Mycelium-based composites (MBCs), produced by cultivating fungal organisms on agricultural or organic waste, offer advantageous properties such as sound absorption, thermal insulation, and resistance to moisture. Nevertheless, their application in architecture and interior design remains constrained, primarily due to concerns regarding aesthetic acceptance [3,4,5]. In interior applications, studies often approach mycelium merely as a substitute for conventional materials rather than focusing on its inherent growth patterns and form-giving capabilities.

The main aim of this research is to investigate how the material potential of mycelium-based biomaterials, which have the capacity to address environmental problems, can be utilized more effectively in interior architecture. In this context, the study adopts a ‘form follows material’ approach, aiming to assess mycelium as a material-specific and environmentally respectful design tool, rather than merely limiting its use as an alternative to conventional materials. This study examines the use of mycelium-based material as an interior component through a digital fabrication approach, focusing on its potential acoustic performance.

The scope of this research covers the process from the conceptual design of an acoustic ceiling component to its physical production and performance evaluation. The study begins with form finding inspired by the structural and textural properties of mycelium, followed by the parametric modeling of a modular component. For the fabrication of the finalized design, molds were created, and preliminary trials using conventional materials were conducted to ensure the molds’ suitability for mycelium production and to optimize their performance. Molds are fabricated using 3D printing technology in three different scales, and mycelium cultivation was carried out under controlled environmental conditions with sterilization measures. In this study, rather than focusing on selecting specific mycelium species and substrates, a commercially available mycelium kit from Biop Biotech was used. This approach was chosen primarily to focus on design variations in mycelium-based composites and to evaluate the resulting products in terms of their acoustic performance and volumetric behavior. Accordingly, the use of a pre-prepared mycelium mixture represents a methodological limitation of the study.

The samples produced are examined in terms of form accuracy, surface texture, and detail resolution through 3D scanning and digital comparison. Finally, acoustic performance is assessed in a modeled interior environment using reverberation time calculations (Sabine method) to determine the potential contribution of the mycelium-based components to spatial acoustic comfort.

The outcomes of this study are expected to provide empirical data on the formability, surface characteristics, dimensional accuracy, and acoustic performance of mycelium-based components produced through digital fabrication techniques, contributing to sustainable design practices in interior architecture.

This paper is organized as follows: Section 1 reviews bio-based materials, mycelium-based composites, and interior applications. Section 2 details the design process, mold design and 3D printing, preliminary plaster and silicone trials, and the setup for mycelium growth. Section 3 presents the mycelium filling and growth, demolding and heat treatment, geometric evaluation via 3D scanning, and the acoustic assessment. Section 4 discusses the study’s achievements in relation to its aims, limitations, implications for future work, and contributions to architectural research, interior practice, and societal benefits.

### 1.1. Bio-Based Materials

Bio-based materials are derived from renewable biological resources and are frequently characterized by high mechanical performance relative to their soft biological constituents [6]. They are considered sustainable because they tend to operate with low energy input and generate minimal waste over their life cycle [7]. Girometta et al. [8] define bio-based composites as materials that contain at least one biologically produced component and are fully biodegradable. Despite originating from soft tissues, such materials can achieve notable strength and mechanical performance, which has attracted attention in architectural and design contexts.

Interest in bio-based materials has grown as part of broader efforts to address long-term environmental challenges, with an increasing number of projects in architecture and design [9]. Research agendas emphasize the development of biologically sourced, recyclable alternatives that could replace conventional materials [10,11]. Within this framing, design can be approached as a living, cyclical system inspired by nature, producing adaptive and regenerative outcomes [12,13]. The study also presents a three-part classification frequently used in the literature—bio-based, bio-responsive, and bio-active materials—which situates mycelium within the bio-based category [6,14].

### 1.2. Mycelium-Based Composites

Mushrooms, which have been part of human life for thousands of years, serve as fundamental components of nature’s digestive system. Due to these inherent traits, they are biologically degradable and emerge as promising constituents for biomaterial designs based on biological cooperation. Mycelium, the vegetative root part of fungi, consists of a dense network of microfilaments called hyphae [15]. Fungi absorb nutrients from their environment through the mycelium, and during this process, the branching of the mycelium merges with organic substrates to form mycelium-based composites with a lightweight, foam-like structure [16]. Throughout the growth process, the mycelium acts as a kind of biological cement by binding the substrate together, while the substrate is partially replaced by the fibrous fungal biomass [8].

Among natural resources that can be cultivated in nature and potentially replace existing construction materials are mycelium-based biocomposites. In recent years, mycelium-based composites have gained attention in architecture and design due to their potential as sustainable building materials, utilizing organic waste streams, requiring minimal energy for production, and naturally decomposing at the end of their life cycle [17]. By developing their mycelial networks, fungi can convert various types of organic waste into composite materials without additional energy input and without generating waste [18]. Mycelium-based composites (MBCs) are typically produced by cultivating fungal mycelium on a lignocellulosic substrate (e.g., sawdust, hemp, flax) where the mycelial network binds the particles into a cohesive structure. Depending on the process, the growth is stopped by drying (reversible dormancy) or heating (permanent inactivation), yielding a composite in which mycelium functions as a natural binder; properties vary with fungal species, substrate composition, growth conditions, and processing [8,16,19].

The study distinguishes Mycelium-Based Materials, which are introduced and classified into two main categories: Mycelium-Based Composites (MBC) and Pure Mycelium Material (PMM). A distinction is drawn between these categories, emphasizing that their properties and potential applications are determined by different parameters [20]. Reported advantages include broad application potential, zero-waste production, and low energy requirements; MBCs also exhibit sound absorption, thermal insulation, and resistance to moisture in use [5]. The ability of mycelial cells to move within porous structures underlies observed self-repair/regeneration behaviors [21]. At the same time, limitations such as relatively low mechanical strength, moisture sensitivity in long-term applications, and variability in surface finish are highlighted in the literature, stimulating work on fiber additions, surface treatment, and improvements in mold/production techniques [16,22,23].

### 1.3. Mycelium in Interior Application and Design Approaches

Mycelium-based composites offer a promising, environmentally friendly alternative for producing sustainable furniture and interior decoration [4]. Designers and architects have increasingly explored mycelium as a sustainable alternative to conventional materials, applying it across a variety of domains, including furniture, interior finishes, and product design [14]. Through natural and spontaneous growth processes, imperfections and irregularities are transformed into aesthetic qualities, resulting in unique material expressions. These characteristics position mycelium as an unconventional and challenging material, where the resulting textures vary depending on the shape of the substrate [24]. In interior contexts, MBCs are primarily positioned for non-structural uses, where their acoustic, thermal, and surface qualities can be exploited without load-bearing demands; contemporary studies, in particular, note their suitability for acoustic applications [5]. The study also notes that many projects continue to replicate conventional architectural elements, thereby restricting the material’s formal potential and underutilizing its growth-driven capacities—an approach that can limit design opportunities [25].

Alongside this critique, this study surveys interior examples that foreground bio-collaborative design and material-led aesthetics—from compostable seating to modular translucent partitions and retail installations—indicating how mycelium can inform both functional and experiential aspects of space (Klarenbeek’s Mycelium Chair; Blast Studio’s Tree Column; Mogu’s Mogu Fields; Studio Aléa’s Back to Dirt; Interesting Times Gang & OBOS’s Veggro; Nina + Co’s BIG Beauty Store; the Belgium Pavilion at the 2023 Venice Biennale; Danielle Trofe & Lujah Brown’s MushLume; Studio Gang’s Populus and the Myco Museum at the 2025 Venice Biennale). Together, these examples frame a trajectory toward modularity, surface expressiveness, and integration with contemporary design workflows. This study presents these entries within its literature/example compilation for interior applications (Table 1).

## 2. Experiment Setup

This section outlines the experimental setup developed for the study, beginning with the design process of the acoustic ceiling component, followed by the mold design and fabrication stages, preliminary trials with conventional materials, and the environmental and sterility preparations for subsequent biological applications.

### 2.1. Design of the Acoustic Ceiling

**Figure 1:**
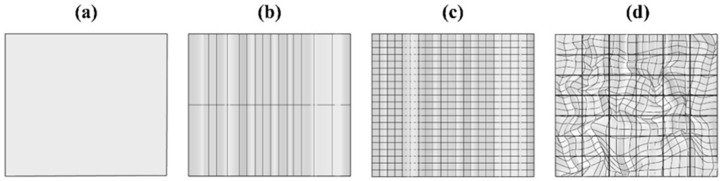
The design process within the Rhinoceros.

The objective of this study is to design an interior acoustic ceiling component that could be fabricated with mycelium-based composites. The formal exploration drew directly on the structural and textural characteristics observed in mycelium, informing both the global surface behavior and local articulation of the component. Within Rhinoceros, the ceiling surface was first organized on a rectangular grid (Figure 1a), divided into grid systems to allow surface deformation (Figure 1b,c), and then subjected to controlled deformation to generate an organic curvature. In this workflow, the Maelstrom tool was used to introduce a rotational twist, while contour lines were extracted to emphasize the surface’s topography and provide guidance for subsequent subdivision. This design logic aimed to enhance acoustic diffusion by increasing surface irregularity across the ceiling plane.

The digitally generated surface was then partitioned into 42 modules (Figure 1d) and arranged across the ceiling field to achieve visual coherence and repeatable assembly logic. Subdivision boundaries followed the expressed topographic lines so that each module captured the underlying curvature rather than flattening it. This ensured that local relief and texture—relevant to acoustic scattering—were preserved at the module scale. The organization of modules also provided a clear interface between design and fabrication, enabling each part to be handled independently in downstream steps.

From a production standpoint, the parametric segmentation established in the design model was conceived to feed directly into mold development and multi-scale fabrication. By keeping the module logic consistent, the same ceiling geometry could be realized at different scales without altering the intended form language. This continuity between digital design and physical making underpinned the iterative, modular mold strategy described in Section 3.2, where detachable faces and multiple scales (1:50, 1:30, 1:15) were adopted to compare form-taking behavior and facilitate demolding.

### 2.2. Mold Design and 3D Printing

The translation of the digital ceiling geometry into a physical, mycelium-compatible mold followed an iterative pathway: each prototype was evaluated and the insights fed back into subsequent revisions to improve demolding, structural stability, and dimensional accuracy. To compare form-taking behavior across sizes, three mold scales were selected: 1:50, 1:30, and 1:15. The molds were conceived as modular units with detachable faces to facilitate demolding and reduce deformation risk. All parts were fabricated with a Bambu Lab A1 3D printer (256 × 256 mm build area) using PLA filament. Early observations underscored the importance of surface integrity and sealing at joints and provided data for later improvements.

The scaling strategy was structured to observe both the overall ceiling system and local geometric behavior. The first mold, printed at 1:30, contained all modules to assess the relationship between the complete design and the interior setting, as well as the joining logic of the modules. The second mold produced the junction of two modules for focused evaluation of connections, while the third captured a single module for detailed observation. Dimensions were specified as follows: 1:50 mold—10 × 10 × 3 cm (depth); 1:30 mold—21 cm (width) × 19 cm (length) × 4 cm (depth); 1:15 mold—9 × 6 × 2 cm. Using multiple scales enabled closer observation of the form’s behavior during physical production.

**Figure 2:**
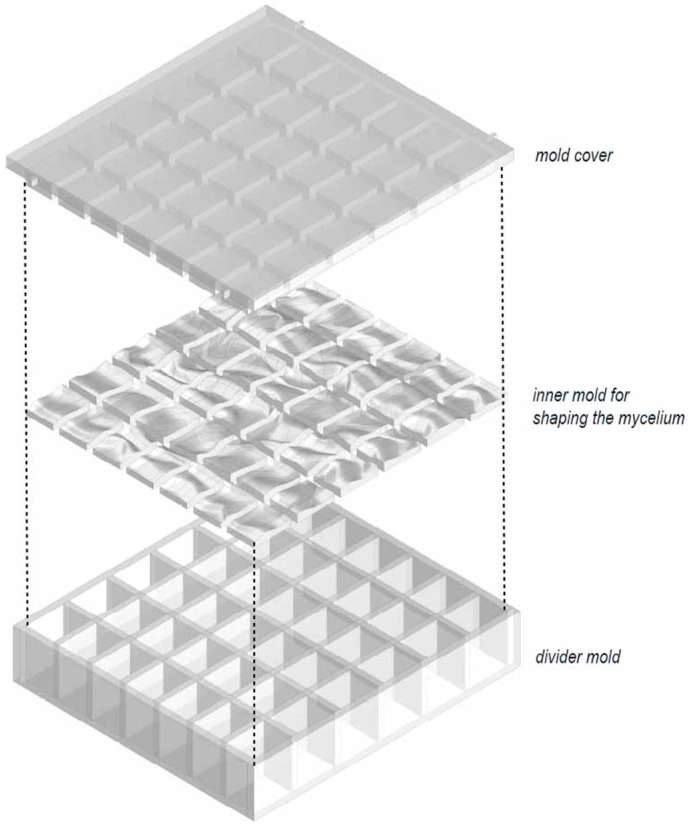
Illustration of the modular mold layers derived from the Rhinoceros design model.

In the final iteration, the mold was designed as a three-part system that allowed each face to be attached and removed independently (Figure 2). This solution was developed after deformation issues were observed in earlier prototypes, with the explicit aim of easing the extraction of the grown piece and improving the functional performance of the mold during demolding.

Additive manufacturing parameters were chosen in line with the available printer and material. The molds were produced on a Bambu Lab A1 (256 × 256 mm build volume) using biodegradable PLA. The study notes PLA’s bio-based origin (commonly derived from corn starch), its biodegradability, and its widespread adoption in 3D printing due to ease of processing and cost efficiency—factors that align with the study’s sustainable material rationale.

Design choices in the mold were also informed by preliminary trials with conventional materials reported in the next subsection. In early plaster casting, the single-piece mold caused the material to wedge inside; the fluid consistency led to leakage through small gaps, and even applying olive oil as a release agent did not prevent breakage during removal—highlighting the need for modular faces and improved sealing. These observations directly fed into the three-part mold solution described above.

Finally, insights gathered here informed downstream biological preparations. As detailed later, separators and a light-blocking lid were introduced to produce smooth side faces, limit light exposure, and apply gentle compression during growth; these elements are implemented in the sterilized growth setup and are referenced here to clarify the continuity between mold design and biological fabrication.

### 2.3. Preliminary Trials with Conventional Materials

#### 2.3.1. Plaster Casting

**Figure 3:**
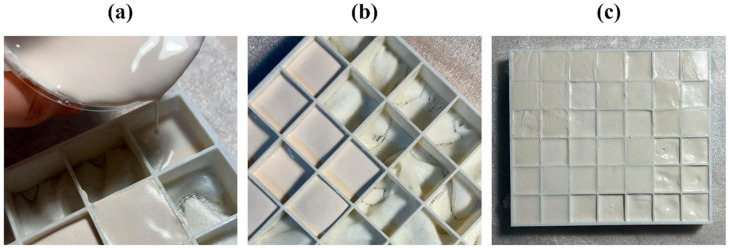
The process of (**a**,**b**) pouring the prepared plaster into (**c**) the mold.

Before working with mycelium, a plaster casting trial was conducted to evaluate how the designed form would behave in a physical mold and to establish a reference for mold–material interaction. Satin plaster, selected for its availability and rapid setting time, was mixed with water and poured into the mold (Figure 3a,b).

Demolding proved problematic due to the mold’s single-piece structure, which caused the plaster to wedge inside. The fluid consistency of plaster also led to leakage through small gaps, preventing homogeneous filling. As shown in Figure 3c, due to leakage from the mold, subsidence occurred in the lower-right area compared to other regions. In a second attempt, olive oil was applied as a release agent, but the plaster still could not be removed without breaking the mold. These results highlighted key design issues—particularly the lack of modularity and surface imperfections—that guided improvements in subsequent iterations.

#### 2.3.2. Silicone Filling

Following the plaster trial, a silicone filling test was performed to assess mold sealing and form-taking capability with a material more similar in consistency to the mycelium substrate. Silicone putty was prepared with starch in an open environment and placed into the molds at all three scales. A rolling pin was used to apply uniform pressure, ensuring the silicone conformed to the mold geometry.

**Figure 4:**
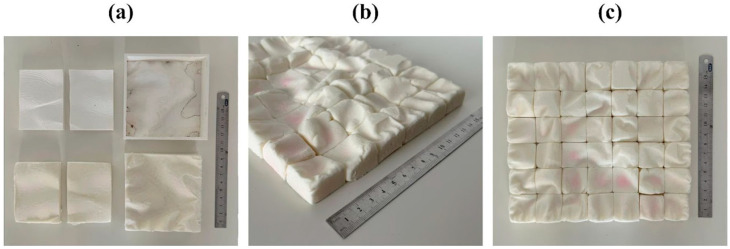
Images of the products obtained from the silicone-filled molds at scales (**a**) 1:15, (**b**) 1:50, and (**c**) 1:30.

This trial confirmed the need for a lid or pressing element to stabilize the geometry during filling and prevent deformation after removal. Although demolding remained somewhat difficult in the non-modular version, silicone’s elasticity allowed removal with minimal shape loss (Figure 4).

### 2.4. Sterilization for the Mycelium Growth

**Figure 5:**
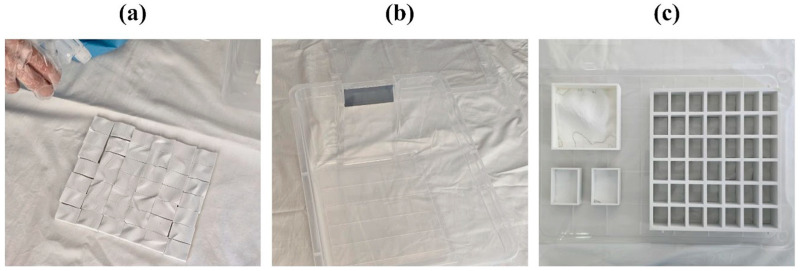
Images of the (**a**) sterilization of the molds, (**b**) light-blocking lid to cover the mold system, and (**c**) placement of the molds into the container.

In preparation for biological experiments, an environmental setup was established to maintain consistent growth conditions. Sterilized molds (Figure 5a) were placed in a transparent, lock-lid container designed to hold three samples simultaneously under identical temperature, humidity, and light conditions. This arrangement minimized contact with the external environment and reduced contamination risks (Figure 5b,c).

Insights from the preliminary trials led to the integration of separators to produce smooth side faces and a light-blocking lid to both cover the mold system and apply gentle compression, ensuring that the material would conform to the intended form during growth.

## 3. Experiments and Results

This section reports the biological applications and their outcomes, proceeding from filling and growth to demolding/heat treatment, geometric evaluation via 3D scanning, and the acoustic assessment.

### 3.1. Mycelium Filling and Growth

**Figure 6:**
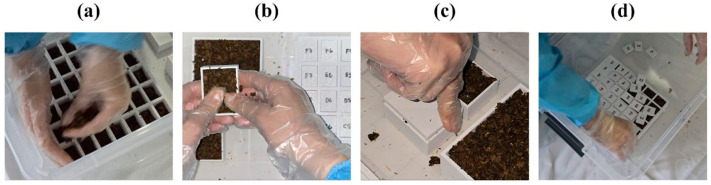
(**a**,**b**) Placement of the mycelium mixture into the molds; (**c**,**d**) Closing of the lids.

For the biological trials, sterilized PLA molds described in Section 3 were filled with a pre-inoculated mycelium–substrate mixture supplied in a semi-solid, moist form (Figure 6a,b). The filling process was performed manually to ensure the mixture was evenly distributed within the cavity, with care taken to press the material into the fine details of the mold geometry. For the purpose of these trials, a ready-to-use mycelium kit from Biop Biotech was employed, allowing the study to focus on the design variations and evaluation of the resulting composite forms rather than on selecting specific fungal species or substrates.

Growth was conducted in a completely dark environment, and a custom light-blocking lid was placed over the mold system to help the mycelium fully conform to the intended geometry and prevent light exposure. We applied gentle compression and separators were placed between the mold faces to create smooth, independent side surfaces (Figure 6c,d). This compression was intended to maintain surface contact during growth and minimize deformation due to expansion. Because the organism develops rapidly and is highly susceptible to contamination, strict sterilization was enforced at every production step; even minor lapses were noted to jeopardize outcomes, underscoring the sensitivity of mycelium fabrication to tightly controlled conditions.

The molds were placed inside a transparent, lock-lid container capable of holding three samples simultaneously. The container was stored in a completely dark environment with stable temperature and humidity to ensure consistent biological activity and to minimize contamination risk.

**Figure 7:**
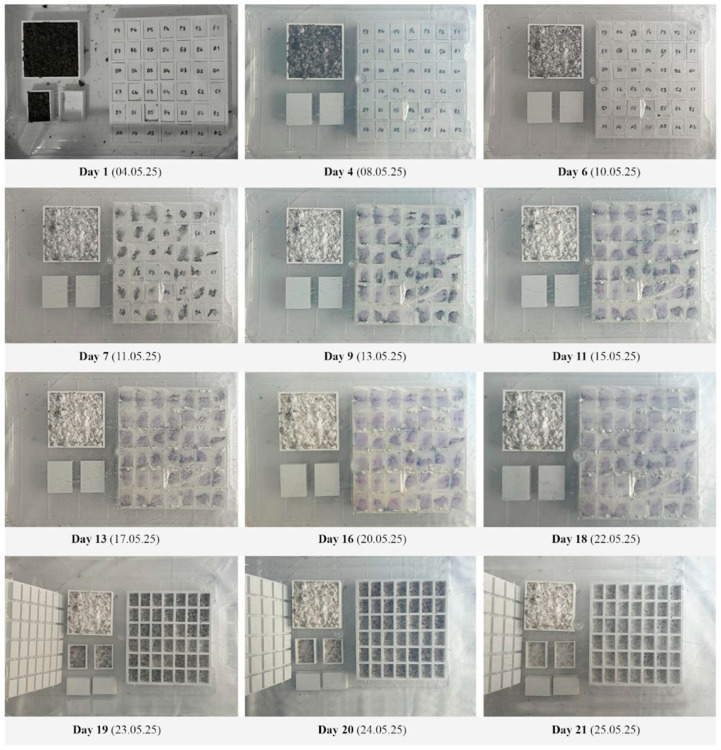
Daily growth stages of the mycelium acoustic ceiling experiment.

To systematically track the progression of mycelial development, the top surface of the 1:50-scale mold was left exposed. This setup enabled a comparative evaluation of mycelial development behavior in both closed and open systems. Throughout the anticipated 21-day incubation period, the stages of growth were regularly photographed and documented (Figure 7). As expected, mycelial growth toward the surface was observed in the open-top mold from the third day onward. This progression was indicated by the emergence of fine, white, web-like hyphal structures on the surface of the soil-like substrate. By the seventh day, traces of mycelial hyphae became more pronounced, and by the ninth day, the surface was largely covered with mycelium, obscuring the markings on the molds. By the ninth day, the mold surface was almost entirely colonized by mycelium.

A key indicator that the mycelium was ready for demolding was the formation of a continuous white layer covering the surface. Based on this criterion, the incubation period was considered complete on the 16th day; however, the molds were retained until the 18th day to monitor any potential variations in growth. At the end of the 18th day, growth was deemed complete, and the molds were removed from the incubation setup.

Over the growth period, the mycelium colonized the substrate, binding particles together into a cohesive, rigid mass. The colonization process gradually replicated the mold’s internal geometry, including the surface texture, though some minor irregularities were visible in areas of high curvature.

### 3.2. Demolding and Heat Treatment

**Figure 8:**
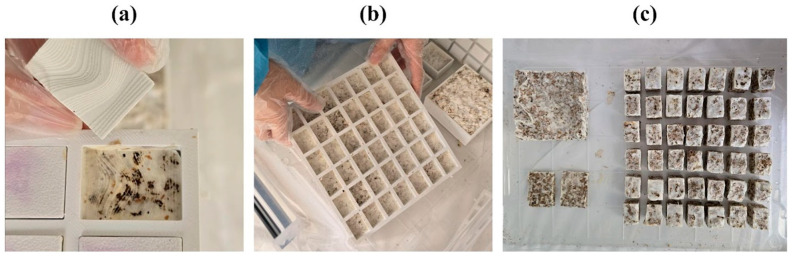
(**a**,**b**) Removal of the mycelium from the mold; (**c**) Placement into a lock-lid container for external drying.

Upon completion of the 18th day of the incubation period, the lids of the molds were removed. In the fully closed molds, the mycelium had largely conformed to the mold geometry; however, high humidity levels were observed, and contrary to expectations, the surface did not fully develop into a uniform white layer or fully harden (Figure 8a,b). To allow homogeneous growth of the mycelium, the lids of the molds were left open, and they were kept under observation in a transparent container for an additional three days.

Once the growth phase was complete, each component was removed from the mold. The modular structure of the mold, combined with the use of separators, significantly reduced the risk of breakage or surface tearing compared to earlier plaster and silicone trials. The modular mold strategy and the dedicated lid not only supported growth but also enabled the removal of the post-growth piece while preserving overall integrity (Figure 8c).

**Figure 9:**
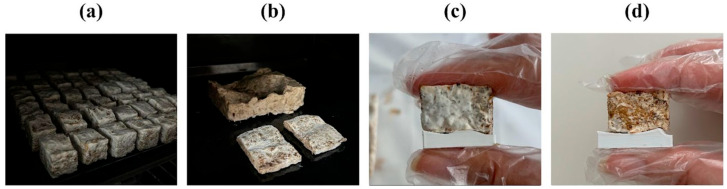
(**a**,**b**) Baking process of the mycelium samples, (**c**) before baking, and (**d**) after baking.

After demolding, the samples were heat-treated at a controlled temperature to inactivate the fungal organism, ensuring the final components were biologically inert (Figure 9a,b). Based on findings from the literature, the termination of mycelial growth was achieved through a gradual oven-drying process. In the first stage, the specimens were maintained at approximately 50 °C for around 180 min to inhibit further fungal proliferation. The duration of the treatment varied according to the size and thickness of the samples, and specimens were periodically monitored until complete dehydration was confirmed. This process significantly reduced the internal moisture content while preserving the structural integrity of the composites. Following drying/heat treatment, moisture loss resulted in a measurable volumetric shrinkage, leading to shape and ratio deviations from the planned model across various scales. The heat treatment also removed residual moisture, contributing to dimensional stabilization. However, shrinkage of varying degrees was observed across all scales (Figure 9c,d).

The finished components largely maintained the intended form and captured the design’s surface articulation, but small deviations appeared in sharper geometric transitions.

### 3.3. Geometric Evaluation by 3D Scanning

**Figure 10:**
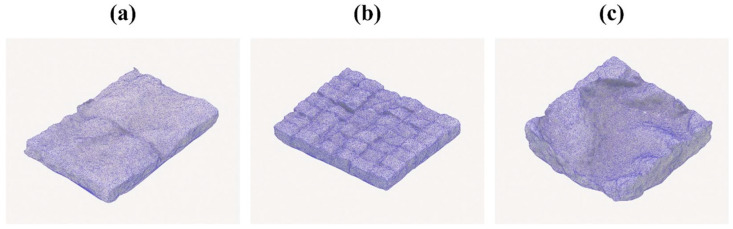
Digital representation of the final scanned products in Polycam at scales of (**a**) 1:15, (**b**) 1:30, and (**c**) 1:50.

The dimensional accuracy and surface fidelity of the produced components were assessed using a workflow combining 3D scanning and computational comparison. Each sample—at scales 1:50, 1:30, and 1:15—was scanned using Polycam to generate a textured 3D mesh (Figure 10). Polycam was selected for its user-friendly interface, cost-free accessibility, and ability to export models in a DAE format compatible with software such as SketchUp 2025. For the 1:30 scale sample, 84 photographs from multiple angles were captured to generate a complete 3D model, which was then exported in DAE format and scaled to 1:1 within SketchUp. The scanned mycelium component was subsequently positioned within the acoustically designed ceiling area of the meeting room for comparative analysis.

These meshes were imported into CloudCompare and aligned with the original CAD models created during the design phase. Due to volumetric shrinkage and moisture loss in the physical samples, minor gaps were observed in the ceiling surface when the scanned models were positioned digitally. The surface areas of both models were calculated in square meters to facilitate subsequent acoustic calculations, with side surfaces included, as the Polycam model was captured as a single piece, which is considered a methodological limitation of the study.

**Figure 11:**
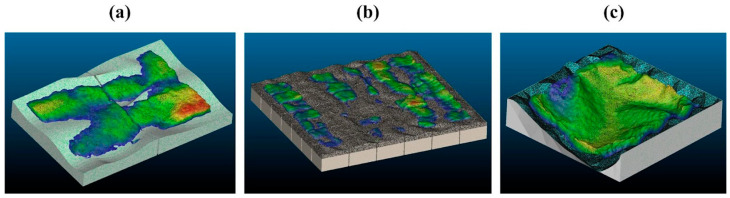
Mycelium-based acoustic ceiling models at scales of (**a**) 1:15, (**b**) 1:30, and (**c**) 1:50, aligned and compared to the final product within the CloudCompare 2.12.2 software.

The Cloud-to-Cloud Distance tool was used to calculate the deviation between the physical sample and the digital reference. This process generated color-coded deviation maps: areas in green indicated close correspondence, while blue and red indicated negative and positive deviations, respectively (Figure 11). The distribution of deviations across the surface was quantitatively demonstrated through analysis of the histogram data obtained from the software.

The 1:30 physical sample was scaled up to 1:1 prior to insertion into the digital meeting-room model. When positioned in the ceiling zone, post-drying shrinkage of the mycelium modules manifested as noticeable gaps between elements.

To quantify the dimensional deviation, the point clouds of the digital and physical models were compared in CloudCompare using a cloud-to-cloud distance analysis. In this method, each point of the physical scan is evaluated against the nearest point on the digital reference surface, and the resulting deviation values are used to determine the matching ratio within a defined tolerance range. The matching rate was calculated by dividing the number of points falling within the defined tolerance by the total number of points in the scanned model.

For each scale, the following parameters were obtained:

- 1:15 scale: Total points = 999,980; points within 0–30 mm tolerance = 759,497 → matching rate: 75.95%;
- 1:30 scale: Total points = 999,980; points within 0–30 mm tolerance = 759,497 → matching rate: 75.91%;
- 1:50 scale: Total points = 1,120,377; points within 0–60 mm tolerance = 850,339 → matching rate: 75.90%.

Directional analysis revealed that the shrinkage was not entirely isotropic; displacement vectors indicated a slightly higher contraction along the vertical (*z*) axis compared to the *x*–*y* plane, most likely due to gravitational influence during the drying phase. Across all samples, post-drying volumetric reduction was evident. The modular mold design helped maintain edge definition and alignment between modules, although slight irregularities remained on complex topographies. Smaller scales generally exhibited better preservation of geometry, especially in areas of fine curvature, whereas larger scales showed more noticeable shrinkage. Across all samples, post-drying volumetric reduction was evident, with dimensional changes concentrated in thinner or unsupported sections of the geometry. The modular mold design helped maintain edge definition and alignment between modules, but slight surface irregularities remained in complex topographies.

### 3.4. Acoustic Performance Assessment

To assess the potential acoustic benefits of the mycelium-based ceiling component, reverberation time (RT) was calculated using the Sabine method, which is the most commonly applied approach for determining sound absorption coefficients in accordance with ISO 354 [36]. The Sabine method calculates reverberation time based on the relationship between the room volume and its total sound absorption, expressed by the equation RT = 0.161·V/ΣA, where V represents the room volume and ΣA the total absorption of all surfaces in sabins. The average reverberation time is measured with and without the test specimen mounted, and the equivalent absorption area (AT) is calculated using Sabine’s equation. This model assumes a perfectly diffuse sound field and a uniform distribution of absorption across all surfaces [36,37,38]. However, for porous and heterogeneous biomaterials, such as mycelium-based composites, the assumptions of uniform absorption and a diffuse sound field may not fully capture the material’s complex acoustic behavior. Nevertheless, the Sabine method remains a standardized and widely accepted approach, and its use in this study is considered sufficient within the scope of comparative evaluation and methodological limitations.

A digital model of a meeting room served as the reference environment, and three acoustic scenarios were simulated:

- Existing without acoustic panel placement condition—the current state of the room without additional acoustic treatment.
- Improved condition with conventional acoustic treatment—application of standard acoustic ceiling panels.
- Prototype condition with the mycelium-based component—integration of the designed mycelium ceiling modules in place of conventional treatment.

The reference environment was a meeting room, a sample meeting room established to assess the spatial performance of mycelium-based acoustic ceiling components. The room was modeled with a 5.0 m ceiling height, 5 × 4.5 m floor dimensions, and a total floor area of 22.5 m^2^, resulting in a volume of 112.5 m^3^. Reverberation time analyses were conducted under an empty-room condition to highlight the room’s acoustic parameters more clearly.

**Figure 12:**
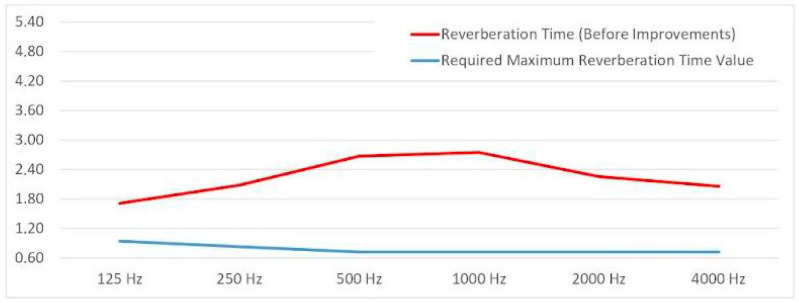
Comparative linear representation of the required maximum reverberation time values by frequency (before improvement).

The Sabine formula was applied using the known absorption coefficients of each material configuration (Table 2). For the mycelium-based panels, the α values were obtained from the manufacturer (Mogu, Inarzo, Italy) based on their in-house testing, providing reliable data for the Sabine formula calculations. The condition without acoustic panel placement exhibited the longest RT, indicating poor speech intelligibility and potential echo problems (Figure 12). The improved condition with conventional panels showed a notable reduction in RT, aligning with typical acoustic comfort standards for meeting spaces. To further contextualize these results, acoustic measurements were conducted across the 125–4000 Hz frequency range, as this spectrum encompasses the majority of human speech frequencies and provides comprehensive information on sound absorption characteristics. Measurements within this frequency range effectively capture variations in absorption across different materials, with higher frequencies generally exhibiting increased sound absorption, making it suitable for a more accurate assessment of speech intelligibility and overall acoustic performance in the studied environment [39].

**Figure 13:**
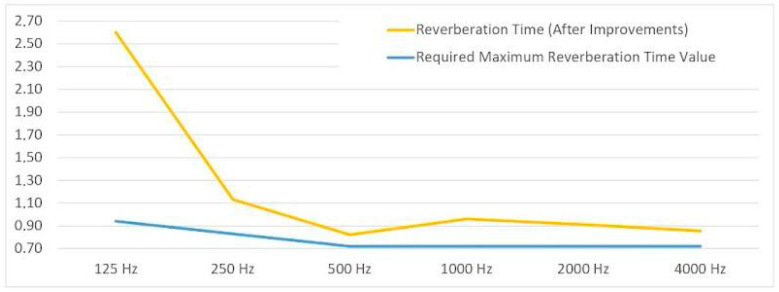
Comparative linear representation of the required maximum reverberation time values by frequency (after improvement).

In the prototype condition, the mycelium-based component produced a measurable reduction in RT compared to the case without acoustic panel placement, approaching the performance of conventional acoustic materials (Table 3). While the prototype did not match the exact absorption performance of standard panels—likely due to smaller coverage area and material thickness—it demonstrated clear potential as a functional acoustic element. The results (Figure 13) confirmed that mycelium-based composites, when integrated into interior architecture, could contribute both aesthetic value and acoustic comfort, with further optimization in material density and surface geometry recommended for future studies.

**Figure 14:**
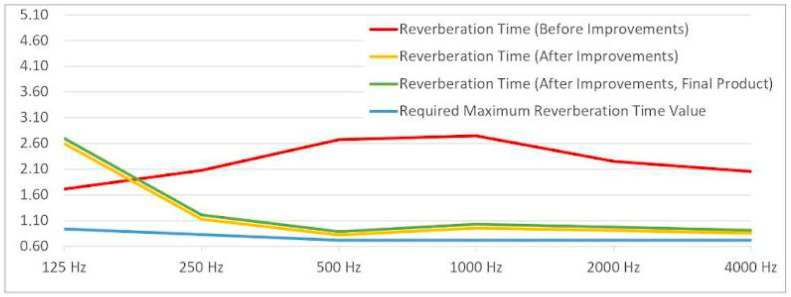
Comparative linear representation of the required maximum reverberation time values by frequency.

In the post-treatment case, reverberation time at 125 Hz was approximately 2.70 s, exceeding the target (0.94 s), whereas at mid–high frequencies (500–4000 Hz) the RT dropped markedly and approached the required 0.72 s maximum. Within 500–1000 Hz—the speech band—the mycelium-ceiling application substantially reduced echo and improved intelligibility, indicating a more balanced and usable acoustic field overall (Table 4, Figure 14).

## 4. Conclusions

This study examined the application of mycelium-based composites (MBCs) as interior architectural components, concentrating on the design, fabrication, and performance evaluation of an acoustic ceiling module. The research was motivated by the need for sustainable, biodegradable, and low-energy alternatives to conventional construction materials, which have high environmental footprints. The primary aim was to investigate how the material potential of mycelium could be effectively utilized in interior architecture, not merely as a substitute for conventional materials but as a design driver informed by its biological growth patterns. Through the design and parametric modeling of a modular acoustic ceiling component, mold fabrication via 3D printing, and subsequent biological growth of pre-inoculated mycelium, this study produced physical prototypes that were evaluated for geometric fidelity and acoustic performance.

The outputs of the experiments demonstrated that MBCs can be shaped into complex, modular geometries with a degree of dimensional accuracy (matching rates of 75–79%) and surface articulation. Acoustic simulations using the Sabine method demonstrated that the mycelium prototype effectively reduced reverberation time compared to spaces without acoustic panel placement, confirming its functional potential as an acoustic element. The integration of parametric design and digital fabrication provided a controllable and repeatable production method.

Several limitations were identified. Mechanical strength and long-term durability under real interior conditions were not tested, leaving behind questions about load-bearing capacity, moisture resistance over time, and fire performance. The acoustic evaluation was simulation-based rather than measured in a real-scale physical environment. Shrinkage and minor surface irregularities occurred after drying, indicating the need for optimization in substrate composition and mold sealing.

Further research could focus on improving the mechanical and physical properties of MBCs by conducting full-scale acoustic measurements and exploring surface pattern optimization for enhanced absorption. Scaling up the fabrication process for larger installations and testing under varying environmental conditions can strengthen the applicability of the material in commercial and public interiors.

This study provides empirical data on the formability, dimensional accuracy, and acoustic potential of MBCs in digitally fabricated modular forms, contributing to the growing body of architectural research on bio-based materials. It bridges material science and computational design by demonstrating a workflow that integrates biological growth processes into parametric form generation.

For interior architects, the findings offer a viable method for incorporating living materials into functional components without sacrificing design flexibility. The use of digital fabrication enables precise control over geometry, modularity for ease of installation, and potential customization for specific spatial acoustics.

On a societal level, adopting materials such as MBCs in the built environment supports the transition toward low-carbon, circular construction practices. The biodegradable nature of mycelium reduces end-of-life waste, while the reliance on agricultural by-products promotes waste valorization. For end-users, interior spaces equipped with mycelium-based acoustic elements can provide improved comfort through sound control, contributing positively to well-being and productivity.

## Figures and Tables

**Table 1:** Mycelium-based composites in interior applications.

No	Project Name/Title	Application Area	Reference
1	Mycelium Chair	Furniture	[26]
2	Tree Column	Partition elements	[27]
3	Mogu Fields	Acoustic wall panels	[28]
4	Back to Dirt	Furniture	[29]
5	Veggro	Decorative surfaces	[30]
6	Big	Furniture	[31]
7	Venice Biennale, Belgium Pavilion	Exhibition design	[32]
8	MushLume	Lighting	[33]
9	Populus	Interior design element	[34]
10	Venice Biennale, Myco Museum	Blocks and wall system	[35]

**Table 2:** Reverberation time calculation results of the meeting room (before improvement).

Properties of Surfaces	Frequency (Hz)												
		125 Hz		250 Hz		500 Hz		1000 Hz		2000 Hz		4000 Hz	
Surface Type	Area (m^2^)	α	S. α	α	S. α	α	S. α	α	S. α	α	S. α	α	S. α
Floor Surface: Linoleum	22.50	0.01	0.23	0.01	0.23	0.01	0.23	0.01	0.45	0.02	0.45	0.02	0.45
Glass	6.40	0.10	0.64	0.13	0.83	0.14	0.89	0.14	1.08	0.18	1.15	0.15	0.96
Ceiling Surface: Painted Plaster	22.50	0.02	0.45	0.02	0.45	0.02	0.45	0.02	0.45	0.02	0.45	0.02	0.45
Wall Surface 1: Painted Plaster	23.40	0.02	0.47	0.02	0.47	0.02	0.47	0.02	0.47	0.02	0.47	0.02	0.47
Wall Surface 2: Painted Plaster	19.14	0.16	3.06	0.14	2.68	0.11	2.11	0.11	1.53	0.08	1.53	0.07	1.34
Wall Surface 3: Painted Plaster	23.40	0.14	3.28	0.10	2.34	0.06	1.40	0.06	1.87	0.10	2.34	0.10	2.34
Wall Surface 4: Painted Plaster	24.63	0.10	2.46	0.07	1.72	0.05	1.23	0.05	0.74	0.02	0.49	0.02	0.49
Door: Wood	3.15	0.00	0.00	0.00	0.00	0.00	0.00	0.00	0.00	0.01	0.03	0.02	0.06
Air (volume)	112.50	0.00	0.00	0.00	0.00	0.00	0.00	0.00	0.00	0.01	1.13	0.02	2.25
Total Absorption, ∑ A (sabins)			10.58		8.71		6.78		6.59		8.03		8.81
Reverberation Time (RT = (0.161 V)/∑ A)			1.71		2.08		2.67		2.75		2.25		2.06
Required Maximum Reverberation Time Value			0.94		0.83		0.72		0.72		0.72		0.72

**Table 3:** Reverberation time calculation results of the meeting room (after improvement).

Properties of Surfaces	Frequency (Hz)												
		125 Hz		250 Hz		500 Hz		1000 Hz		2000 Hz		4000 Hz	
Surface Type	Area (m^2^)	α	S. α	α	S. α	α	S. α	α	S. α	α	S. α	α	S. α
Floor Surface: Linoleum	22.50	0.02	0.45	0.02	0.45	0.03	0.68	0.04	0.90	0.04	0.90	0.05	1.13
Glass	6.40	0.10	0.64	0.07	0.45	0.05	0.32	0.03	0.19	0.02	0.13	0.02	0.13
Ceiling Surface: Mycelium	31.30	0.10	3.13	0.40	12.50	0.60	18.76	0.50	15.63	0.50	15.63	0.50	15.63
Wall Surface 1: Painted Plaster	23.40	0.02	0.47	0.02	0.47	0.02	0.47	0.02	0.47	0.02	0.47	0.02	0.47
Wall Surface 2: Painted Plaster	19.14	0.02	0.38	0.02	0.38	0.02	0.38	0.02	0.38	0.02	0.38	0.02	0.38
Wall Surface 3: Painted Plaster	23.40	0.02	0.47	0.02	0.47	0.02	0.47	0.02	0.47	0.02	0.47	0.02	0.47
Wall Surface 4: Painted Plaster	24.63	0.02	0.49	0.02	0.49	0.02	0.49	0.02	0.49	0.02	0.49	0.02	0.49
Door: Wood	3.15	0.30	0.95	0.25	0.79	0.15	0.47	0.10	0.32	0.10	0.32	0.07	0.22
Air (volume)	112.50	0.00	0.00	0.00	0.00	0.00	0.00	0.00	0.00	0.01	1.13	0.02	2.25
Total Absorption, ∑ A (sabins)			6.97		16.00		22.03		18.85		19.91		21.16
Reverberation Time (RT = (0.161 V)/∑ A)			2.60		1.13		0.82		0.96		0.91		0.86
Required Maximum Reverberation Time Value			0.94		0.83		0.72		0.72		0.72		0.72

**Table 4:** Reverberation time calculation results of the meeting room (after improvement, final product).

Properties of Surfaces	Frequency (Hz)												
		125 Hz		250 Hz		500 Hz		1000 Hz		2000 Hz		4000 Hz	
Surface Type	Area (m^2^)	α	S. α	α	S. α	α	S. α	α	S. α	α	S. α	α	S. α
Floor Surface: Linoleum	22.50	0.02	0.45	0.02	0.45	0.03	0.68	0.04	0.90	0.04	0.90	0.05	1.13
Glass	6.40	0.10	0.64	0.07	0.45	0.05	0.32	0.03	0.19	0.02	0.13	0.02	0.13
Ceiling Surface: Mycelium	28.60	0.10	2.86	0.40	11.45	0.60	17.17	0.50	14.31	0.50	14.31	0.50	14.31
Wall Surface 1: Painted Plaster	23.40	0.02	0.47	0.02	0.47	0.02	0.47	0.02	0.47	0.02	0.47	0.02	0.47
Wall Surface 2: Painted Plaster	19.14	0.02	0.38	0.02	0.38	0.02	0.38	0.02	0.38	0.02	0.38	0.02	0.38
Wall Surface 3: Painted Plaster	23.40	0.02	0.47	0.02	0.47	0.02	0.47	0.02	0.47	0.02	0.47	0.02	0.47
Wall Surface 4: Painted Plaster	24.63	0.02	0.49	0.02	0.49	0.02	0.49	0.02	0.49	0.02	0.49	0.02	0.49
Door: Wood	3.15	0.30	0.95	0.25	0.79	0.15	0.47	0.10	0.32	0.10	0.32	0.07	0.22
Air (volume)	112.50	0.00	0.00	0.00	0.00	0.00	0.00	0.00	0.00	0.01	1.13	0.02	2.25
Total Absorption, ∑ A (sabins)			6.71		14.94		20.45		17.53		18.59		19.84
Reverberation Time (RT = (0.161 V)/∑ A)			2.70		1.21		0.89		1.03		0.97		0.91
Required Maximum Reverberation Time Value			0.94		0.83		0.72		0.72		0.72		0.72

## Data Availability

The original contributions presented in this study are included in the article. Further inquiries can be directed to the corresponding author.
